# Development of a Saw Bones Model for training pedicle screw placement in scoliosis

**DOI:** 10.1186/s13104-017-3029-3

**Published:** 2017-12-04

**Authors:** Gregory Tanner, Saman Vojdani, David E. Komatsu, James M. Barsi

**Affiliations:** Stony Brook University Hospital, Stony Brook, USA

**Keywords:** Saw bones, Surgical simulation, Scoliosis, Pedicle screw

## Abstract

**Objective:**

The purpose of this study was to determine if a Sawbones Scoliosis Model could be used as a simulator to train residents in placing pedicle screws—a complex procedure with a steep learning curve. Surgical simulation, a common tool teaching residents complex procedures in a safe environment, was staged using a Sawbones Scoliosis Model. Ten junior and ten senior residents out of 25 total possible residents (80%) were instructed how to place pedicle screws using the free-hand technique. They were then asked to place them unilaterally from T4 to L4 and were assessed on completion time, accuracy placement accuracy, and overall competency using an objective rating scale.

**Results:**

Senior residents had an average time to completion of 38.9 ± 4.7 min vs. junior’s 50.1 ± 11.7 min, and a pedicle screw accuracy of 43.6 ± 6.4% vs. junior’s 44.4 ± 17.4%. Overall competency scores were similar for both groups; however, senior residents scored higher in the time and motion subdomain. Senior residents had a faster completion time and were more efficient, suggesting greater experience in spine surgery. The low rate of screw accuracy in both groups validates that simulation is a safe way for trainees to learn complex tasks.

## Introduction

Complex surgical procedures are often described as having a learning curve where technique improves with experience. One such procedure is placement of pedicle screws in the treatment of scoliosis [1–5]. With knowledge of spinal anatomy and surface landmarks, pedicle screws can be placed safely using the free hand technique described by Lenke [6, 7]. The rate of screw misplacement has been characterized as 14–25% [8, 9]. Screw malposition has the potential for catastrophic neurologic and vascular complications [10]. While placement of pedicle screws remains a complex procedure best left to experienced surgeons, orthopaedic surgical residents must learn this technique during their training.

Historically, surgical education included trainees performing procedures on real patients while attending surgeons supervised. With the ever-increasing complexity of procedures, this method of training may not prove safe or provide maximal educational benefit.

Simulation as an educational tool for complex procedures has been used in other industries, such as airlines, with much success. Only recently has surgical simulation been developed to train surgical residents. In orthopaedics, simulators have been described in arthroscopy, fracture surgery and joint arthroplasty [11–14]. Pedicle screw simulation has been described before, but not with a Scoliosis Model [15, 16]. Idiopathic scoliosis is associated with dysplastic pedicle morphology and a complex three-dimensional deformity that makes pedicle screw placement more challenging [17, 18].

The purpose of this study is to validate a Scoliosis Training Model as a simulation technique in training residents how to place pedicle screws. Our hypothesis is that senior residents will have a faster completion time, pedicle screw accuracy rate, and higher score on an objective rating scale compared to junior residents.

## Main text

### Methods

This project was reviewed by the institutional review board and determined to be exempt.

#### The model

A Scoliosis Spine Model (Model 1323021; Sawbones, Vashon, Washington) comprising of T1 to the sacrum was used for the simulation. This model has a 55° right thoracic curve with an associated 25° rotation. A scoliosis spine holder (Model 1703-100; Sawbones, Vashon, Washington) was used to secure the model during instrument insertion. To simulate surrounding musculature, the model was supported in the holder by polystyrene foam peanuts and the model secured in place up to the level of the transverse processes. Peanuts were also inserted between the spinous processes and vertebral foramen in order to prevent direct visual assistance of insertion of the pedicle or spinal cord.

#### Subjects and simulation

Ten senior (fourth and fifth year) and ten junior (first, second, or third year) residents from a US orthopaedic residency program, out of a possible 25 total residents (80%), were recruited for this study. Participation in the study was voluntary.

Prior to beginning the simulation, the residents were given verbal instructions describing the simulation by a fellowship trained orthopaedic surgeon. The free hand technique for placement of pedicle screws was explained in detail. The subjects were randomized either the right or left side of the spine. Pedicle screws were placed from T4 to L4, and each participant was instructed to place one screw at a time. The hand dominance of the subjects was recorded. All required instruments and pedicle screws (Medtronic, Memphis, TN) were laid out next to the model. Subjects were timed from start to task completion. Videos of the simulation were delivered to a blinded, experienced pediatric spinal deformity surgeon for review. A checklist of steps and global rating scale (Table 1) was developed for this exercise. To prevent bias, the videos were cropped so as only to only show the model and the hands of the subject. The global scale used is a modified version previously validated in the literature [19–21].

**Table 1:** Objective rating scale

	1	2	3	4	5
Preparation	Did not organize, has to stop procedure frequently to prepare equipment		Equipment generally organized		All equipment neatly prepared and ready for use
	1	2	3	4	5
Time and motion	Many unnecessary movements		Efficient time and motion		Clear economy of movement and maximum efficiency
	1	2	3	4	5
Instrument handling	Repeatedly makes tentative or awkward moves with instruments		Competent use of instruments, occasionally appeared stiff and awkward		Fluid moves with instruments and no awkwardness
	1	2	3	4	5
Flow of procedure	Frequently stopped procedure and seemed unsure of next moves		Demonstrated some forward planning with reasonable progression of procedure		Obviously planned course of procedure with effortless flow from one move to the next
	1	2	3	4	5
Knowledge of procedure	Deficient knowledge		Knew all important steps		Demonstrated familiarity with all aspects of the procedure
	1	2	3	4	5
Overall performance	Very poor		Competent		Clearly superior

Grading rubric for steps and global rating scale for resident performance

After insertion of screw from T4 to L4, each model was photographed before removing the implants. Each screw was evaluated for accuracy, and misplaced screws were recorded as to their location. Average screw length and diameter were obtained. The final results of screw placement were recorded and shared with the blinded reviewer.

#### Statistical analyses

For the statistical comparisons, data from post-graduate year (PGY) 4 and 5 residents were grouped as seniors, and data from PGY1, 2, and 3 residents were grouped as juniors. The data were then presented as group mean ± standard deviation. An f test was performed using Excel (Microsoft, Redmond, WA) to assess data normality with multiple outcomes found to be non-normally distributed. As such, significant differences between groups were assessed using Mann–Whitney tests in SPSS (Version 19, SAS Institute, Cary, NC). For all tests, p values less than 0.05 were considered significant.

### Results

**Fig. 1:**
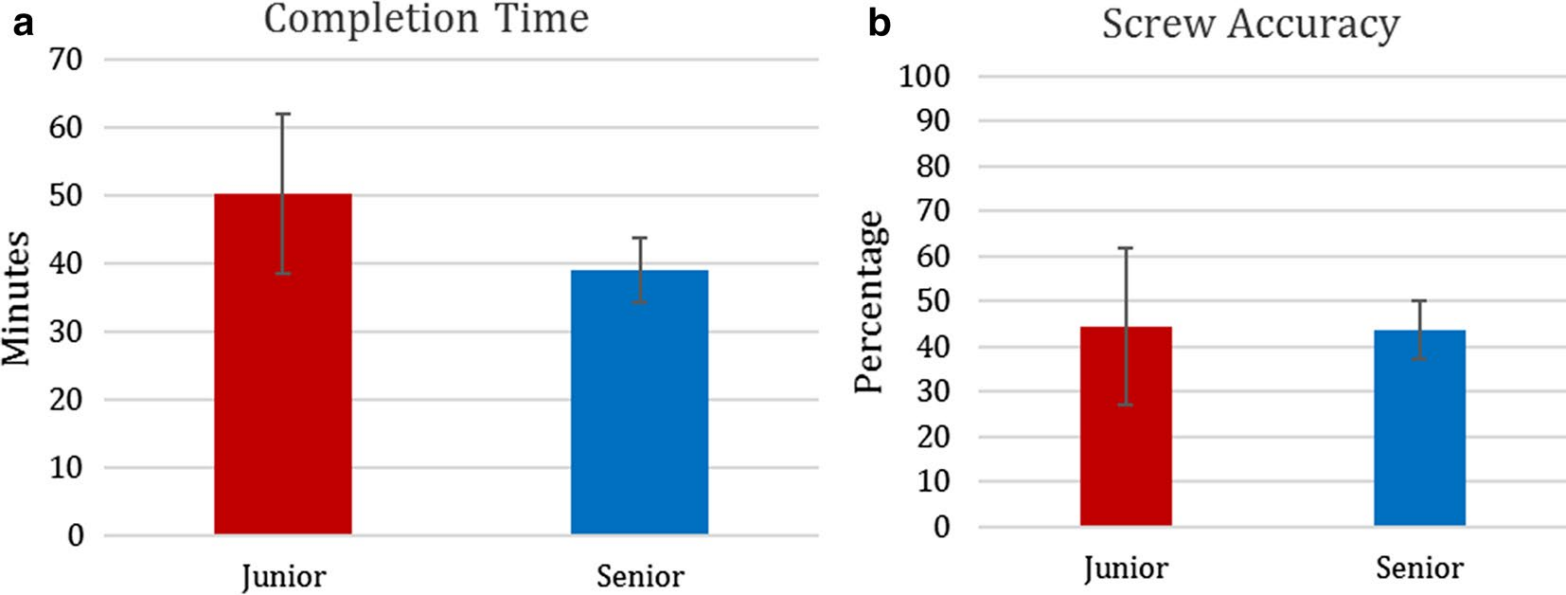
**a** Completion time in minutes for junior and senior residents. **b** Screw accuracy for junior and senior residents. Graphic illustrating comparative group findings

Completion time was 50.1 ± 11.7 min for junior residents and 38.9 ± 4.7 min for senior residents, revealing a significant increase in speed for the senior residents (p = 0.018) (Fig. 1a). Screw accuracy defined as the pedicle screw being completely within the pedicle was 44.4 ± 17.4% for junior residents and 43.6 ± 6.4% for senior residents, demonstrating no significant differences in accuracy (p = 0.847) (Fig. 1b). The objective global rating scale showed no overall difference between junior and senior residents; however, there was a statistically significant difference in the time and motion subdomain (Table 2).

**Table 2:** Scores on objective global exam

	Junior residents	Senior residents	p value
Preparation	3.29 ± 0.49	3.43 ± 0.53	0.591
Time and motion	2.71 ± 0.49	3.71 ± 0.95	*0.030*
Instrument handling	2.86 ± 0.90	3.14 ± 0.69	0.496
Flow	3.29 ± 0.49	3.57 ± 0.53	0.298
Knowledge	3.29 ± 0.49	3.71 ± 0.76	0.244
Overall	3.29 ± 0.49	3.57 ± 0.53	0.298

Compiled scores for residents based on rubric. This table presents the mean ± standard deviation for each of the five evaluated surgical skills demonstrated by the junior and senior residents. p values obtained from Mann–Whitney tests comparing the groups are presented in the last column with significant values (i.e., p > 0.05) highlighted in italics

### Discussion

A common dilemma among those who educate residents is how to impart procedural knowledge to the trainee. Historically, resident education used an apprenticeship model. With the increasing complexity of procedures and breadth of knowledge required of residents during their surgical education, educational tools have been developed to enhance the learning experience.

Simulation of the patient encounter has been the standard in medical student education. Only recently has simulation of complex procedures been developed. Simulation as an educational tool has been given paramount importance as the American Board of Orthopaedic Surgery in concert with the Accreditation Council for Graduate Medical Education mandated the development of a surgical skills curriculum for orthopaedic interns.

With increasing complexity of a procedure, so increases the corresponding learning curve. Pedicle screw placement in idiopathic scoliosis is one such complex procedure. The combination of a three dimensional deformity along with dysplastic anatomy make this a difficult procedure even in experienced hands. In order to teach this skill in a safe setting that had no associated risk to real patients, a Scoliosis Model was tested.

As we expected, senior residents, who have had more surgical experience and training, had a faster completion time for the task. On the objective rating scale, senior residents had more economy of motion compared to less experienced trainees.

The finding of low screw accuracy rate in both groups suggests that placement of pedicle screws in scoliosis remains an extremely challenging task. Most orthopaedic surgeons who place pedicle screws in practice have completed advanced fellowship training. Given the high malposition rate and the associated risk of injury of misplacement, the effectiveness of simulation of this technique becomes evident. Lieberman et al. showed that robotic guidance was able to improve the accuracy of pedicle screw placement in a cadaveric study of normal spines [16], but our simulation may be able to offer similar improvements without the costs associated with guidance systems. Similarly, Luciano et al., studied the ability of a haptic feedback system to improve pedicle screw placement [15], but did not find a significant effect for their training. Combined with our study, these studies show there are many options for instructing residents in pedicle screw placement in a safe environment without any associated patient risk. Moreover, using the tools outlined in our study, the experienced surgeon can use objective measures to assess the trainee in any of these modalities.

### Limitations

The limitations of this study include selection bias since it was limited to trainees at a single institution. Residency programs are unique in their curriculum development. Including institutions with more emphasize on orthopaedic spine surgery might have altered the results. Another bias is the very nature of simulation. Even though attempts were made to make this simulation feel as real as possible, nothing can substitute for the operative experience on live patients. Finally, we did not have preliminary data to run a power analysis to establish a proper sample size. Due to these limitations, other institutions should weight the merits of our simulation against others before adopting any specific training program. However, it is our intent to further develop this training program as validate protocol for improving the accuracy of pedicle screw placement are needed. Further studies will confirm if the skills learned in this exercise are transferable to real clinical scenarios.

In conclusion, a Sawbones Scoliosis Model can be used as an educational tool in teaching orthopaedic residents how to perform the complex task of placing pedicle screws. Senior residents, who had more surgical experience, scored higher on the objective measures of completion time and economy of motion subdomain on a global rating scale. However, given the limitations of this study, other residency programs will have to decide if this simulation is appropriate for their training goals.

## Abbreviations

US: United States
T4: 4th thoracic vertebrae
L4: 4th lumbar vertebrae
PGY: post-graduate year
SPSS: Statistical Package for the Social Sciences
Min: minutes
